# Functional consequence of the *MET-T*1010I polymorphism in breast cancer

**DOI:** 10.18632/oncotarget.3094

**Published:** 2014-12-31

**Authors:** Shuying Liu, Funda Meric-Bernstam, Napa Parinyanitikul, Bailiang Wang, Agda K. Eterovic, Xiaofeng Zheng, Mihai Gagea, Mariana Chavez-MacGregor, Naoto T. Ueno, Xiudong Lei, Wanding Zhou, Lakshmy Nair, Debu Tripathy, Powel H. Brown, Gabriel N. Hortobagyi, Ken Chen, John Mendelsohn, Gordon B. Mills, Ana M. Gonzalez-Angulo

**Affiliations:** ^1^ Department of Breast Medical Oncology, The University of Texas MD Anderson Cancer Center, Houston, TX, USA; ^2^ Department of Systems Biology, The University of Texas MD Anderson Cancer Center, Houston, TX, USA; ^3^ Department of Investigational Cancer Therapeutics, The University of Texas MD Anderson Cancer Center, Houston, TX, USA; ^4^ Department of Bioinformatics, The University of Texas MD Anderson Cancer Center, Houston, TX, USA; ^5^ Department of Veterinary Medicine, The University of Texas MD Anderson Cancer Center, Houston, TX, USA; ^6^ Section of Breast Cancer Translational Research, Department of Breast Medical Oncology, The University of Texas MD Anderson Cancer Center, Houston, TX, USA; ^7^ Department of Biostatistics, The University of Texas MD Anderson Cancer Center, Houston, TX, USA; ^8^ Department of Clinical Cancer Prevention, The University of Texas MD Anderson Cancer Center, Houston, TX, USA; ^9^ Department of Experimental Therapeutics, The University of Texas MD Anderson Cancer Center, Houston, TX, USA

**Keywords:** MET mutations, Breast Cancer, Malignant transformation

## Abstract

Major breast cancer predisposition genes, only account for approximately 30% of high-risk breast cancer families and only explain 15% of breast cancer familial relative risk. The HGF growth factor receptor MET is potentially functionally altered due to an uncommon germline single nucleotide polymorphism (SNP), *MET*-T1010I, in many cancer lineages including breast cancer where the *MET-*T1010I SNP is present in 2% of patients with metastatic breast cancer. Expression of *MET-*T1010I in the context of mammary epithelium increases colony formation, cell migration and invasion *in-vitro* and tumor growth and invasion *in-vivo*. A selective effect of *MET-*T1010I as compared to wild type MET on cell invasion both *in-vitro* and *in-viv*o suggests that the *MET-*T1010I SNP may alter tumor pathophysiology and should be considered as a potential biomarker when implementing MET targeted clinical trials.

## INTRODUCTION

A strong breast cancer family history can signal the presence of inherited risk-modifying genetic events. However, mutations in *BRCA1/2*, the major breast cancer predisposition genes, only account for approximately 30% of high-risk breast cancer families and only explain 15% of breast cancer familial relative risk [1-5]. Even including the estimated contributions of mutations in high penetrance (*BRCA1/2, TP53, CDH1, LKB1,* and *PTEN*), and moderate or low penetrance genes including SNPs associated with breast cancer identified through genome-wide association studies (GWAS), 50% of familial breast cancer predisposition still remains unexplained [6].

The hepatocyte growth factor (HGF) and its receptor, the transmembrane tyrosine kinase MET, promote cell proliferation, survival, motility, and invasion, as well as morphogenic changes that can stimulate repair and regeneration in normal tissues. The greatest effects of HGF, also known as scatter factor, are most clearly manifest through cellular motility and invasion. As with many growth factor receptors, the actions of MET are co-opted during tumor growth [7]. MET over-expression, with or without gene amplification has been reported in a variety of human malignancies [7-10] where it has been suggested to provide a major resistance mechanism for targeted therapies against multiple receptors [7]. Non-synonymous germ-line mutations in the kinase domain of MET were initially described in patients with hereditary papillary renal carcinoma [11]. Sporadic and germline MET mutations have since been detected in multiple solid tumors. However, only a subset of these mutant alleles have been proven to cause malignant transformation providing a potential for therapeutic targeting [12]. In many cases, the impact of germline and somatic aberrations remains controversial and is likely context dependent with different effects based on intrinsic lineage expression programs or mutational context in particular cancer lineages.

Here we report that the *MET*-T1010I germline mutation occurs in approximately 2% of patients with metastatic breast cancer. Overexpression of wild type *MET* (*MET*-WT) as well as expression of *MET-*T1010I increases colony formation, cell migration and invasion *in-vitro* and tumor growth *in-vivo*. A selective effect of *MET-*T1010I on cell invasion both *in-vitro* and *in-vivo* as compared to *MET*-WT suggests that *MET-*T1010I may alter pathophysiology and should be considered for inclusion in clinical trials of MET inhibitors.

## RESULTS

**Figure 1:**
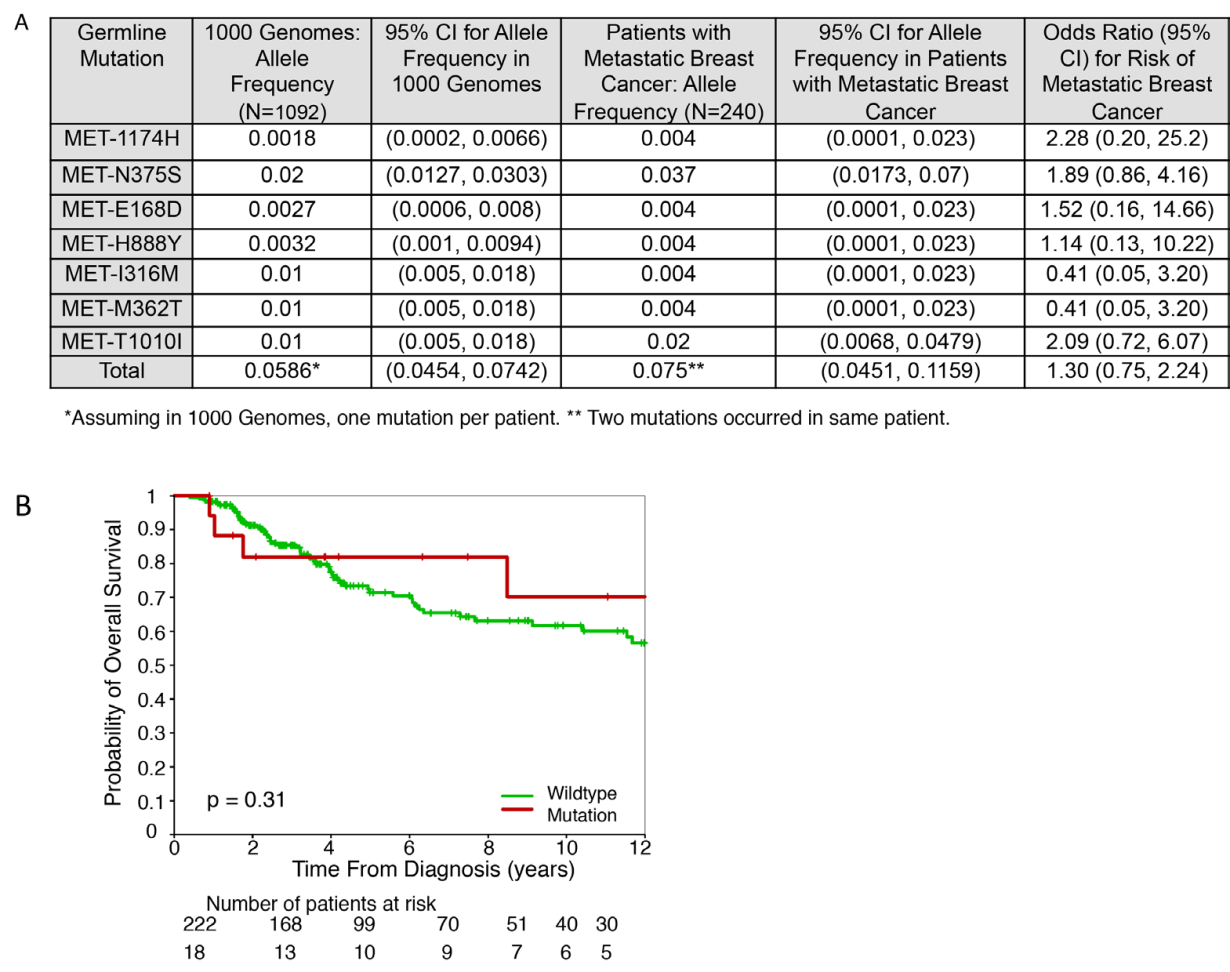
Germline mutations and survival estimates in metastatic breast cancer Tumor and blood samples were obtained under an Institutional Review Board-approved prospective collection protocol at MD Anderson Cancer Center (MDACC). After informed consent, patients with metastatic breast cancer underwent biopsy of their metastatic disease and blood collection. For genomic DNA library preparation, and target capture, whole exome sequencing and data analysis, we called single nucleotide variants (SNVs) and small indels using an in-house developed analysis pipeline based on variant allele frequencies in the tumor and the matched normal tissues. (A) Germline *MET* mutations detected in patients with metastatic breast cancer, and their frequencies as compared with normal population (1000 Genome Project). (B) Overall survival outcomes of patients with *MET* germline mutations versus patients with wild type *MET.*

Matched tumor tissue and blood from 240 patients with metastatic breast cancer were submitted for targeted exome sequencing. Three somatic mutations (*MET*-M1192I, *MET*-L269V, and *MET*-E34K) were found in three different patients. We also found 19 germline mutations in 18 patients (one patient had two germline mutations). Figure 1A shows the *MET* germline mutations detected in patients with metastatic breast cancer, and their frequencies as compared with a normal population with a similar ethnic mix (1000 Genomes Project, www.1000genomes.org). Two recurrent germline mutations (as these SNPs are potentially functional) *MET-*N375S (n = 9) and *MET-*T1010I (n = 5) were observed. The frequency of *MET*-T1010I in patients with metastatic breast cancer was twice that in the general population (Odds Ratio [OR] = 2.09, 95% CI: 0.72−6.07). Based on the odds ratio of 2.0, we decided to assess the functional consequence of *MET*-T1010I in breast cancer models. Table 1 shows the patient characteristics assessed by *MET* germline mutation status. At a median follow-up of 44 months (range, 5−384 months), there were 69 (28.7%) deaths. Five-year OS was 82% in *MET* mutation carriers and 71% in patients without *MET* mutations, (*P* = 0.18), respectively (Figure 1B). These are not significantly different from the outcomes for patients without germline aberrations but are limited by the low number of events in the MET mutation population.

**Table 1:** Patient and tumor characteristics

	All Patients (N = 240)	***MET*** Germline Wildtype (N = 222)		***MET*** Germline Mutation (N = 18)		
	N (%)	N	%	N	%	P
Age, years						
Age ≤ 50	163(67.9%)	151	68.0	12	66.7	
Age > 50	77(32.1%)	71	32.0	6	33.3	0.90
Nuclear grade						
I	6(2.6%)	5	2.4	1	5.6	
II	63(27.4%)	59	27.8	4	22.2	
III	161(70%)	148	69.8	13	72.2	0.42*
Subtype						
Hormonal positive	114(59.1%)	104	58.4	10	66.7	
HER2 positive	25(13%)	25	14.0	0	0.0	
Triple negative	54(28%)	49	27.5	5	33.3	0.36*
Lymphovascular Invasion						
Negative	114(52.3%)	99	49.5	15	83.3	
Positive	104(47.7%)	101	50.5	3	16.7	0.006*
Neoadjuvant therapy						
No	165(68.8%)	150	67.6	15	83.3	
Yes	75(31.3%)	72	32.4	3	16.7	0.19*
Adjuvant Therapy						
No	71(29.6%)	67	30.2	4	22.2	
Yes	169(70.4%)	155	69.8	14	77.8	0.59*

* Fisher's exact p-value.

**Figure 2:**
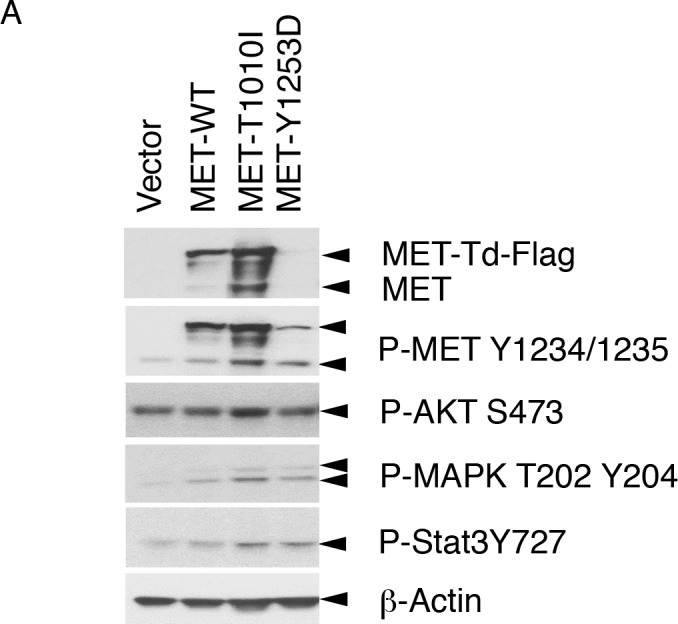

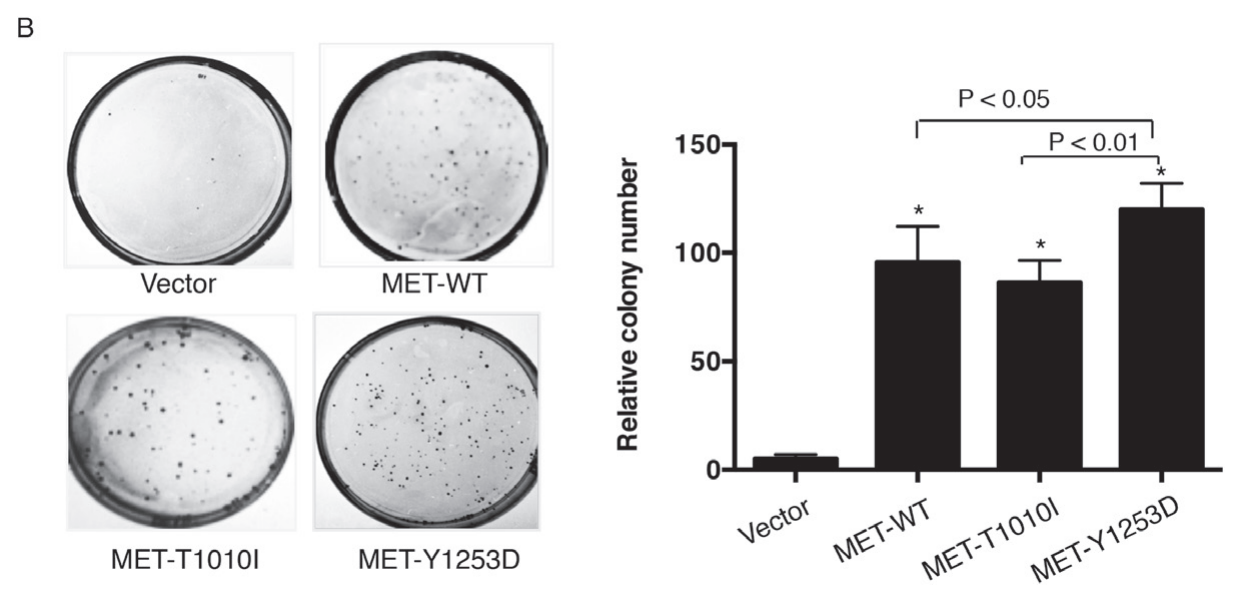

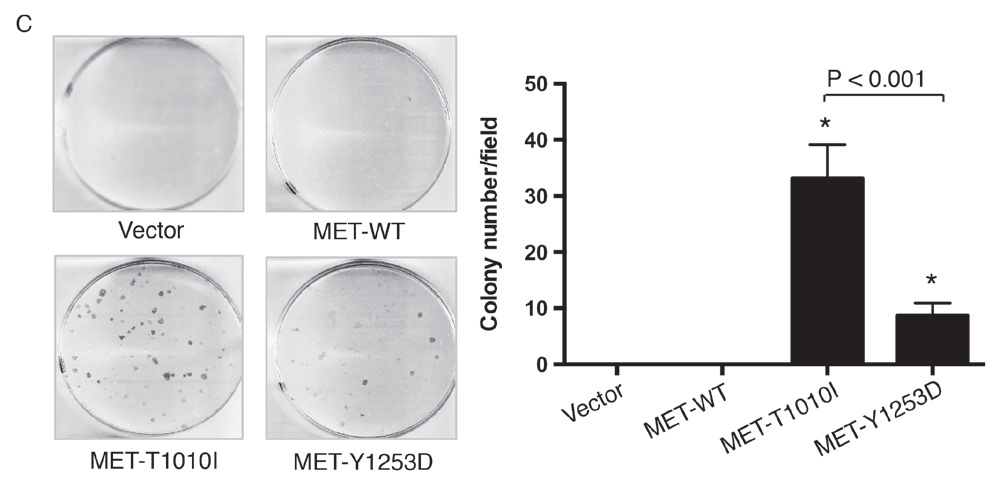

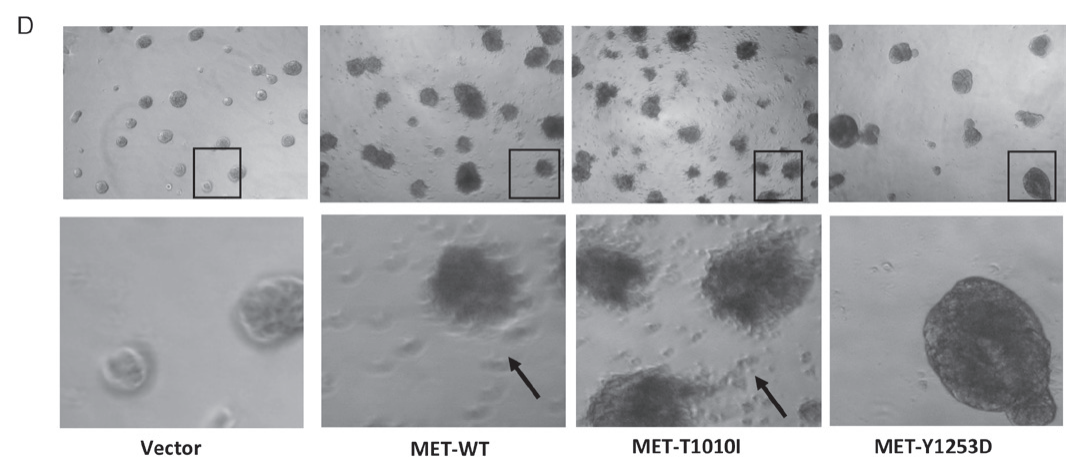

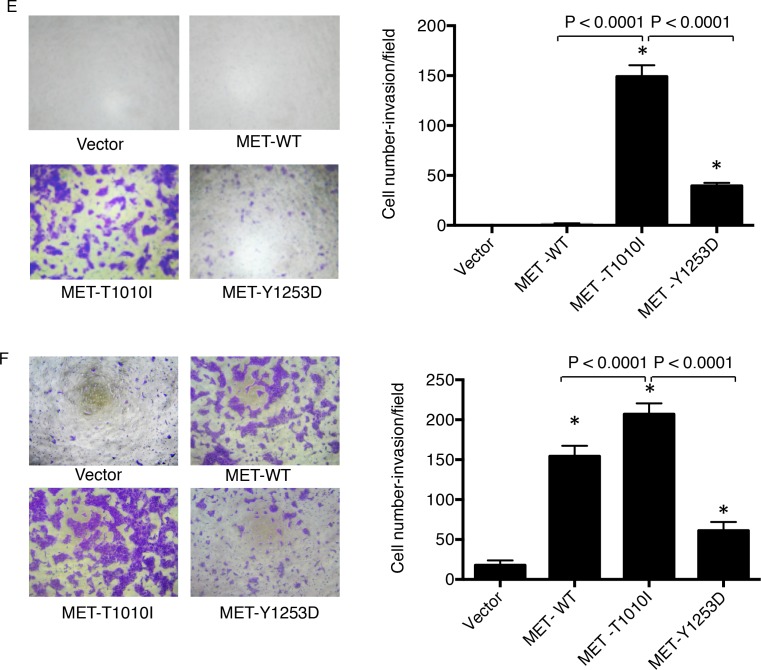
Transforming effects of *MET* aberrations in breast epithelial cells (A) Effects of *MET* aberrations on cell signaling: Lysates of MCF-10A derived cells (as indicated) were used for Western blot. Effects of *MET* aberrations on colony formation were tested as described in Materials and Methods. MCF-10A derived cells (as indicated) were seeded in triplicate. Cells were cultured in 2.5% horse serum, lacking EGF and insulin, with HGF (40 ng/ml) (B) or without HGF (C). Photos were taken at day 11. Data are mean ± standard error of triplicates, representative of two independent experiments (*, *P* < 0.0001 versus Vector). ANOVA. (D) Effects of MET aberrations on mammary acinar morphogenesis were tested as described in Materials and Methods. MCF-10A derived cells (as indicated) were resuspended in modified growth medium containing 2% matrigel, 2% horse serum, and 5 ng/mL EGF, supplemented with HGF 40 ng/ml. Representative field images of acini were taken on day 8; original magnification, X40. Effects of *MET* overexpression or mutations on cell invasion were tested as described in Materials and Methods. MCF-10A derived cells (as indicated) were induced with fibronectin (5 μg/ml) alone (E) or both HGF (40 ng/ml) and fibronectin (5 μg/ml) (F). Cells were photographed at X100 magnification. Data are mean ± standard errors of triplicates, representative of two independent experiments. (*, *P* < 0.0001 versus Vector). ANOVA.

To determine if *MET*-T1010I alters breast cancer pathophysiology, we compared its effects to *MET*-WT representing MET overexpression in breast cancer and to *MET*-Y1253D tyrosine kinase domain mutation representing an activated MET receptor. First, to evaluate the effects of *MET* aberrations on cell signaling, we tested the expression and phosphorylation levels of endogenous and exogenous MET and its downstream targets, including phosphorylation of AKT, MAPK and STAT3 with Western blotting (Figure 2A). *MET*-WT and *MET*-T1010I were markedly overexpressed with *MET*-T1010I expressed to slightly higher levels than *MET*-WT potentially due to decreased turnover due to interference with the effects of Y1003-mediated phosphorylation and degradation [13]. In contrast *MET*-Y1253D was expressed to modest levels potentially due to increased internalization and degradation of activated MET receptors [13]. Under basal conditions, all three constructs demonstrated increased MET phosophorylation (pY1234/1235) consistent with constitutive activation. Under basal conditions, *MET* transfected cells, regardless of the construct, demonstrated a modest but consistent increase in phophorylation of MAPK consistent with activation of the MET receptor. There was a slight increase in phosphorylation of AKT and STAT3 with the *MET*-T1010I construct compared to the other constructs.

To investigate whether *MET*-T1010I affects cell survival, we performed clonogenic assays. In the presence of HGF, *MET*-WT and the two mutants (T1010I, Y1253D) markedly increased colony formation and colony size compared to control cells in low serum medium in the absence of EGF and insulin which are required for optimal proliferation of MCF-10A (Figure 2B). Interestingly, both *MET* mutants, but not *MET*-WT or control, induced colony formation in the absence of HGF, with *MET*-T1010I inducing more and larger colonies than *MET*-Y1253D (Figure 2C), suggesting that MET mutants are less dependent on HGF than WT MET. In a three-dimensional Matrigel culture system in the presence of HGF, MCF-10A cells expressing *MET*-T1010I formed larger acini with abnormal structures, scattering to the surrounding matrix (Figure 2D). WT MET cells showed similar but milder morphological alterations. In contrast, cells expressing *MET*-Y1253D did not demonstrate detectable invasion into Matrigel. In contrast, *MET*-Y1253D cells had normal structure but formed larger acini than control cells, suggesting that mutant *MET* (*MET*-T1010I) and overexpression of WT MET in the presence of HGF promote both cell proliferation and invasion in Matrigel, whereas *MET*-Y1253D increased cell proliferation but not invasion. However, in a Boyden chamber invasion assay, even in the absence of HGF, both *MET* mutations (T1010I and Y1253D) were sufficient to significantly increase invasion (*P* < 0.0001), with the T1010I mutant exhibiting greater invasive capacity than Y1253D (*P* < 0.0001) (Figure 2E). Further, *MET*-WT increased invasiveness in the presence of HGF (Figure 2F).

**Figure 3:**
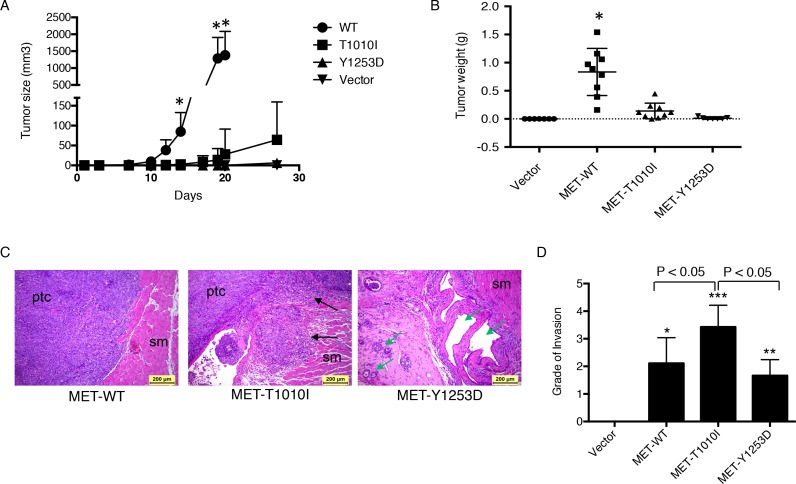
Effects of MET aberrations on MCF-10A xenograft in hHGF transgenic mice (A) A total of 1×10^7^ MCF-10A derived cells (as indicated) were injected into the mammary fat pad of hHGF/SCID females. Each group consisted of 7-9 mice. Tumor volume was calculated with the formula (*V* = *lw*^*2*^/2) (Mean ± SEM; ANOVA, *, *P* < 0.05 MET-WT versus T1010I, Y1253D and Vector groups, respectively). (B) Tumor weight. (*, *P* < 0.05 compared with T1010I, Y1253D and Vector groups, respectively). ANOVA. (C) H&E for histological images from representative tumor from *MET*-WT, *MET*-T1010I and *MET*-Y1253D. All tumors from the *MET*-WT and *MET*-T1010I groups showed dense cellularity with undifferentiated and markedly pleomorphic tumor cells (ptc), and high mitotic rate. In contrast, tumors from *MET*-Y1253D had significantly lower cellularity with less pleomorphic and well-differentiated tumor cells forming glandular and acinar structures (green arrows). Compared to tumors from the *MET*-WT group, *MET*-T1010I tumors exhibited more aggressive invasion (black arrows) into surrounding adipose tissue and skeletal muscle (sm). (D) Data are presented as grade of tumor invasion (*, *P* < 0.05; **, *P* < 0.01; ***, *P* < 0.0001 versus Vector). ANOVA.

To determine whether MET overexpression or mutation was sufficient to induce tumor formation and invasion *in-vivo*, we established tumor xenograft models in human HGF transgenic mice on a severe combined immunodeficiency (SCID) background, named hHGF Tg SCID [14]. As expected, vector-transfected MCF-10A cells did not form tumors. Furthermore, only one hHGF negative mouse of 5 injected with *MET*-T1010I expressing tumor cells developed a tumor and none of the *MET*-WT or *MET*-Y1253D cohorts developed tumors indicating that human HGF is required for optimal tumor development in SCID mice. Tumors grew in all mice in the *MET*-WT group (9/9) and most mice in the *MET*-T1010I (7/9) cohort with 8- and 12-day latent periods respectively, while only 3/7 mice in the *MET*-Y1253D cohort formed tumors which were both much smaller and had a longer latent period (20 days) (Figure 3A). Tumor weight in *MET*-WT group was higher than that in *MET*-T1010 and *MET*-Y1253D groups (*P* < 0.0001, respectively) (Figure 3B).

Xenograft tumors and all organs of each mouse were processed into hematoxylin and eosin (H&E) stained sections, which were examined and evaluated histopathologically by a veterinary pathologist blinded to the genotype of the tumors. Tumors were evaluated histologically (cellular density and morphology, mitotic rate, and invasion into surrounding tissues), and all other organs were examined for the presence of tumor metastasis. All tumors from the *MET*-WT and *MET*-T1010I groups showed dense cellularity with undifferentiated and markedly pleomorphic tumor cells (ptc), and high mitotic rate. In contrast, tumors from *MET*-Y1253D had significantly lower cellularity with less pleomorphic and more well-differentiated tumor cells forming glandular or acinar structures (green arrows). Compared to tumors from the *MET*-WT group, *MET*-T1010I tumors exhibited more aggressive tumor invasion (black arrows) into surrounding skeletal muscle (sm) (Figure 3C). Local or peripheral infiltration of tumor cells into surrounding adipose, skeletal muscle or mammary gland tissues were graded into 5 levels (0−4) by a veterinary pathologist (Figure 3D). Consistent with the *in-vitro* data, *MET*-T1010I tumors exhibited aggressive invasion into surrounding tissues. No metastasis was detected at the 28-day time-point in any group.

## DISCUSSION

We demonstrate that *MET* germline mutations occur in 7% of patients with metastatic breast cancer with *MET*-T1010I found in 2% of patients. This was not detected in the Collaborative Oncological Gene-environment Study (COGS) potentially due to our study being restricted to patients with metastatic breast cancer [4,6]. More recently, Kurian et al. performed targeted exon sequencing of 42 genes including *MET* in the germline DNA of 198 women referred for *BRCA* testing, 174 with breast cancer. This study of limited size also failed to detect germline mutations in *MET* [15]. In contrast, the *MET*-T1010I SNP has been reported to be associated with hereditary renal papillary cancer, non-small cell lung cancer, colon cancer, and gastric cancer and has been found in a patient with breast cancer [12,16-18]. In our study, germline *MET*-T1010I mutations are in excess of the numbers predicted based on allele frequency in the 1000 Genomes Project in metastatic breast cancer, however, due to the low number of cases, this did not reach significance.

We did not detect survival differences between *MET* mutation carriers and non-carriers in this small sample set. In survival studies looking at the prognostic effect of *BRCA*1/2 mutation in breast cancer, no OS differences have been demonstrated. However, *BRCA* mutations have been associated with longer recurrence-free survival in triple-negative disease [19]. However, larger studies will be required to determine whether these SNPs in *MET* are associated with altered patient outcomes.

Somatic and germline mutations in *MET* are predominantly located in the non-kinase domain, mainly in regions encoding the extracellular semaphorin domain (E168D, L229F, S323G, and N375S) and the intracellular juxtamembrane domain (R988C, T1010I, S1058P, and exon-14 deletions) [20]. However, only some of these mutant alleles have been proven to cause malignant transformation as a result of constitutive receptor activation posing a potential for therapeutic targeting or altering response to therapy targeting other signaling molecules [12]. The *MET* juxtamembrane domain regulates ligand-dependent MET internalization as a consequence of Y1003 phosphorylation in response to HGF binding leading to MET ubiquitination and degradation [13]. Mutations in the juxtamembrane domain such as T1010I have been proposed to result in MET accumulation at the cell surface and persistent signaling leading to tumorigenic activity [13]. Indeed in our studies when expressed in MCF-10A as compared to a constitutively active MET variant, *MET*-T1010I is expressed at an elevated level potentially contributing to its selective effect on invasion *in-vitro* and *in-vivo*. Though some previous reports suggested that *MET*-T1010I could have oncogenic properties, the results of functional studies are contradictory. Schmidt et al. proposed that the T1010I mutation is a SNP as it lacked the ability to transform NIH-3T3 cells [11]. Tyner et al. found similar results in the BA/F3 murine myeloid cell model [21]. In contrast, Lee et al. found tumorigenic effects in their NIH-3T3 model [17], and Tengs et al. reported the transforming potential of *MET*-T1010I in non-small cell lung cancer [22]. We demonstrate that *MET*-T1010I has marked functional consequences in the MCF-10A normal breast epithelial cell line, which is more likely to reflect the appropriate context for breast cancer than previous studies with BA/F3 and NIH-3T3. Interestingly, our data showed differences between effects of the somatic activating *MET* mutation, *MET*-Y1253D, and the germline *MET*-T1010I. Despite a low level of expression in MCF-10A compared to *MET*-WT, *MET*-Y1253D increased cell survival independent of exogenous HGF *in vitro*, suggesting that increased kinase activity is sufficient for these processes. However, in contradistinction to *MET*-WT and *MET*-Y1253D, *MET*-T1010I selectively increases invasion both *in-vitro* and *in-vivo*. This effect of *MET*-T1010I is not fully recapitulated by an activating mutation in the tyrosine kinase domain or by overexpression of *MET*-WT suggesting that the effects of *MET*-T1010I are not solely due to overexpression or due to increased kinase activity. Whether this selective effect relates to increased *MET*-T1010I stability [13] will require further exploration.

Exogenous expression of *MET*-T1010I resulted in a modest increase in expression of exogenous MET-Flag and endogenous MET compared to the other constructs. This was associated with a slight increase in basal phosphorylation of MET, MAPK, AKT and STAT3. However, these differences compared to *MET*-WT or *MET*-Y1253D were modest. As indicated above, decreased levels of *MET*-Y1253D in MCF-10A cells are consistent with phosphorylation-mediated degradation [23]. Since the juxtamembrane domain regulates ligand-dependent MET internalization as a consequence Y1003 phosphorylation leading to MET ubiquitination and degradation [13], the *MET*-T1010I SNP in the juxtamembrane domains may contribute to MET accumulation at the cell surface and persistent pathway activation contributing to effects on tumor pathophysiology [13].

In conclusion, we found *MET* germline mutations in 7% of patients with metastatic breast cancer, with at least one of the SNPs contributing to aggressive behavior when expressed in the MCF-10A cell model. Our findings constitute evidence justifying further studies of the frequency of *MET* germline mutations in high-risk populations, the role of MET inhibitors in cancer prevention, and the use of *MET*-T1010I as biomarker for inclusion of patients in therapeutic studies of MET inhibitors.

## METHODS

### Human tissues

### Clinical information

Tumor and blood samples were obtained under a prospective collection protocol at MD Anderson Cancer Center (MDACC). After informed consent, patients with metastatic breast cancer underwent biopsy of their metastatic disease and blood collection. Patients and tumor characteristics were collected by chart review. The Institutional Review Board of MDACC approved the laboratory study. Patients with metastatic breast cancer were categorized according to *MET* germline mutations (mutant versus wild type). Patients' characteristics including age, race, tumor size, lymph nodes, pathologic stage, histology, grade, lymphovascular invasion, and (neo)adjuvant chemotherapy were tabulated and compared between groups using Fisher's exact test. Overall survival (OS) was measured from the date of diagnosis to the date of death or lost to follow-up. The Kaplan-Meier product limit method was used to estimate the survival outcomes of all patients by groups and was compared using the log-rank statistic.

### Genomic DNA and library preparation

Genomic DNA was quantified by Qubit (Invitrogen) and quality was accessed using Genomic DNA Tape for the 2200 Tapestation (Agilent). DNA from each sample (200-500 ng of genomic DNA) was sheared by sonication with the following conditions: Peak Incident Power 175, Duty Cycle 20%, Intensity 5, Cycles per Burst 200, and 120 seconds using Covaris E220 instrument (Covaris). To ensure the proper fragment size, samples were checked on TapeStation using the DNA High Sensitivity kit (Agilent). The sheared DNA proceeded to library prep using KAPA library prep kit (KAPA) following the “with beads” manufacturer protocol. Briefly, this protocol consists of 3 enzymatic reactions for end repair, A-tailing and BioO adaptor ligation, followed by barcode (BioO) insertion by PCR using KAPA HiFi polymerase (6 cycles). PCR primers were removed by using 1.8x volume of Agencourt AMPure PCR Purification kit (Agencourt Bioscience Corporation). At the end of the library prep, samples were analyzed on TapeStation to verify correct fragment size and to ensure the absence of extra bands. Samples were quantified using KAPA qPCR quantification kit. Equimolar amounts of DNA were pooled for capture (8-12 samples per pool).

### Targeted capture

202 genes including *MET* that are clinically relevant in cancer, based on mutational data in the Catalogue of Somatic Mutations in Cancer (COSMIC) and The Cancer Genome Atlas (TCGA) with a minimum of 3% frequency across disease sites or 5% disease specific were selected for capture. We designed biotin labeled probes with Roche Nimblegen for capturing target regions (all exons in those 202 genes) and followed manufacture's protocol for the capture step. Briefly, DNA was pooled (8-16 samples), dried out and after addition of the capture reagents and probes; samples were incubated at 47^o^C on thermocycler with heated lid (57^o^C) for 64−74 hours. The targeted regions were recovered using streptavidin beads and the streptavidin-biotin-probe-target complex was washed and another round of PCR amplification was performed according to manufacturer's protocol. The quality of each captured sample was analyzed on TapeStation using the DNA High Sensitivity kit and the enrichment was accessed by qPCR using specific primers designed by Roche Nimblegen. The cutoff for the enrichment was 50-fold minimum.

### Sequencing

The captured libraries were sequenced on a HiSeq 2000 (Illumina Inc., San Diego, CA, USA) on a version 3 TruSeq paired end flowcell according to manufacturer's instructions at a cluster density between 700 –1000 K clusters/mm^2^. Sequencing was performed on a HiSeq 2000 for 2 × 100 paired end reads with a 7 nt read for indexes using Cycle Sequencing v3 reagents (Illumina). The resulting BCL files containing the sequence data were converted into “.fastq.gz” files and individual libraries within the samples were demultiplexed using CASAVA 1.8.2 with no mismatches. All regions were covered by >20 reads.

### Data Analysis

We aligned the T200 target-capture deep-sequencing data to human reference assembly GRCh37 using BWA [24] and removed duplicated reads using Picard [25]. We called single nucleotide variants (SNVs) and small indels using an in-house developed analysis pipeline [26], which classified variants into 3 categories: somatic, germline, and loss of heterozygosity based on variant allele frequencies in the tumor and the matched normal tissues. We called copy number alterations using a previously published algorithm [27], which reports gain or loss status of each exon. To understand the potential driver identity and functional consequence of detected variants, we compared them with dbSNP, COSMIC [28], and TCGA databases, and annotated them using VEP [29], ANNOVAR [30] and CanDrA [31].

### Cell lines and animal models

### Construction of recombinant lentiviruses expressing wild type and mutant MET

GeneART synthesized constructs expressing human wild type (WT) or mutant *METs* (*MET*-WT-Flag, *MET*-T1010I-Flag, and *MET*-Y1253D-Flag) were designed by us. pMA vector 2 was used as the backbone in these plasmids. The constructs were sequenced to ensure the validity of the sequence and the orientation. Then the human full-length cDNAs for wild type *MET* and the two mutants, with Kozak sequence before ATG and Flag-tag after *MET*, were sub-cloned into pLVX-tdTomato-N1 vectors (Clontech, Mountain View, CA) with XhoI/Xmal enzymes. The constructs (pLVX-*MET*-WT-Flag-tdTomato, pLVX-*MET*-T1010T-Flag-tdTomato, pLVX-*MET*-Flag-Y1253D and the empty vector pLVX-tdTomato-N1) were sequenced by GeneART to confirm the sequence. We confirmed that the orientation of the constructs was correct with restriction enzymes.

### Generation of Lentiviruese expressing wild type and mutant METs

To generate the lentiviruses expressing wild type *MET* and its mutants, we used two expression systems. One of them was the ViraPower Lentiviral Expression System (Invitrogen). In addition, we used the Lenti-X^TM^ Lentiviral Expression System (Clontech). A total amount of 7 μg of pLVX-tdTomato-N1 vector, pLVX-*MET* WT-Flag-tdTomato, pLVX-*MET* T1010T-Flag-tdTomato, or pLVX-*MET*-Y1253D-Flag-tdTomato were co-transfected into the Lenti-X 293T cells, with 36 μg of the Lenti-X HTX packaging Mix, using 7.5 μl Xfect Polymer (Clontech, CA, USA). Lentiviruses containing supernatants were collected after 48 hours, followed by a brief centrifugation (500 g for 10 minutes) and passed through a 0.45 μm filter to remove cellular debris. Then they were used to infect the mammary epithelial cells. Selection began 48 hours after infection in growth medium with 1 μg/ml puromycin for two weeks. Both lentiviral expression systems allowed similar specific expression levels.

### Cell culture

MCF-10A, non-transformed mammary epithelial cell lines obtained from our Characterized Cell Line Core, MDACC and grown at 37°C in humidified 5% CO_2_. MCF-10A cells were maintained in DMEM/F12 (Thermo Scientific, South Logan, Utah) supplemented with 5% horse serum (Invitrogen), 20 ng/ml EGF (Peprotech), 10 μg/ml insulin (Sigma), 100 ng/ml cholera toxin (Sigma), 0.5 μg/ml hydrocortisone (Sigma), 100 units/ml penicillin and 100 μg/ml streptomycin. Cells were frozen at early passages and used for less than 4 weeks in continuous culture.

### Western Analyses

Cells were washed twice with cold phosphate-buffered saline and lysed in ice-cold lysis buffer [1% Triton X-100, 50mM HEPES, pH 7.<4, 150mM NaCl, 1.5mM MgCl_2_, 1mM EGTA, 100mM NaF, 10mM Na pyrophosphate, 1mM Na_3_VO_4_, 10% glycerol, protease inhibitor cocktail (Roche Applied Science), and phosphatase inhibitors, PhosSTOP (Roche Applied Science)]. Western blot was performed as described previously [32].

### Morphogenesis Assay

Three-dimensional culture of cells was carried out on a Matrigel basement membrane [33]. Briefly, 4 x10^3^ cells were resuspended in a modified growth medium, containing 2% growth factor-reduced Matrigel (BD Biosciences), and subsequently seeded onto the Matrigel matrix in 8-well chamber slides (BD Bioscience). Medium was replaced every 3 days. Photographs of representative fields were taken as indicated. Acini were photographed and counted in 10 randomly chosen fields and expressed as means of triplicates, representative of three independent experiments.

### *In vitro* Invasion Assay

Cell *in-vitro* invasion was analyzed with 24-well Biocoat Matrigel invasion chambers with 8 μm polycarbonated filters (Becton Dickinson). Cells were starved for 20 hours in serum-free DMEM F12 lacking growth factors. A total of 1×10^5^ cells in 0.6 ml DMEM F12 were inoculated into the upper chamber, and 0.75 ml DMEM F12 containing fibronectin (5 μg/ml) with or without HGF (40 ng/ml) was added to the lower chamber. The cells were allowed to pass through the Matrigel at 37°C, 5% CO_2_, for 22 hours. Non-invasive cells on the upper surface of the filter were removed by wiping with a cotton swab. The cells that penetrated through the pores of the Matrigel to the underside of the filter were stained with 0.25% crystal violet in 20% methanol for 30 minutes. Invasive cells were photographed and counted in 10 random fields.

### Clonogenic Assay

For clonogenic assays, 1,000 cells were seeded in a 60 mm dish, in growth medium, for 11 days. For inhibitory assay, after attaching on the dish, cells were treated for 2 days with drugs as designed. The cells were rinsed with PBS, followed by staining with 0.25% crystal violet / 20% ethanol. Quantitative analysis of the total number and size of clones was performed with AlphaVIEW SA software (Cell Biosciences).

### Tumor Xenografts studies

All animal studies were carried out under ACUF-approved protocols. hHGF Tg SCID females or hHGF negative littermates at 6-8 weeks of age were used. Exponentially growing MCF-10A derived cells, including *MET*-WT, *MET*-T1010I, *MET*-Y1253D, and control cells (vector), were harvested. After being washed, 1×10^7^ cells in growth factor reduced Matrigel and PBS (1:1), in total 200 μl, were injected into the mammary fat pads of mice. Each group consisted of 7-9 mice. Tumor sizes were determined by measuring the length (*l*) and the width (*w*) with calipers twice weekly. Tumor volume was calculated with the formula (*V* = *lw*^2^/2). Differences in tumor volume among groups at each time point were analyzed using ANOVA. At the end of the experiment (28 days after tumor cell injection), mice were sacrificed. Tumors were harvested, followed by measurement of tumor size and weight. Tumors were cut and flash-frozen in liquid nitrogen for Western blot or fixed in 10% neutral-buffered formalin for histopathological analysis. Fixed tissues were processed, embedded in paraffin, and cut in 4 μm thick tissue sections that were stained with H&E for microscopic examination. Xenograft tumors and all the organs, of each mouse, were subjected to double-blind histopathological analysis by a veterinary pathologist.

### Statistical Analyses

Statistical analyses were carried out using the ANOVA test (for multiple groups) and the Student *t* test (for two groups). Differences with *P-*values < 0.05 were considered statistically significant.
